# Agent-Based Semantic Role Mining for Intelligent Access Control in Multi-Domain Collaborative Applications of Smart Cities

**DOI:** 10.3390/s21134253

**Published:** 2021-06-22

**Authors:** Rubina Ghazal, Ahmad Kamran Malik, Basit Raza, Nauman Qadeer, Nafees Qamar, Sajal Bhatia

**Affiliations:** 1 Department of Computer Science, COMSATS University Islamabad (CUI), Islamabad 45550, Pakistan; rubinaghazal@uaar.edu.pk (R.G.); basit.raza@comsats.edu.pk (B.R.); 2 University Institute of Information Technology, PMAS Arid Agriculture University Rawalpindi, Rawalpindi 46300, Pakistan; 3 Department of Computer Science, Federal Urdu University of Arts, Science &Technology, Islamabad 45570, Pakistan; nauman.qadeer@fuuast.edu.pk; 4 Department of Health Administration, Governors State University, Chicago’s Southland, University Park, IL 60484, USA; mqamar@govst.edu; 5 School of Computer Science and Engineering, Sacred Heart University, 3135 Easton Turnpike, Fairfield, CT 06825, USA; bhatias@sacredheart.edu

**Keywords:** access control, intelligent RBAC, multi-domain collaboration, dynamic environments, smart city applications, semantic role mining, ontology, multi-agent system, word embedding, LSTM

## Abstract

Significance and popularity of Role-Based Access Control (RBAC) is inevitable; however, its application is highly challenging in multi-domain collaborative smart city environments. The reason is its limitations in adapting the dynamically changing information of users, tasks, access policies and resources in such applications. It also does not incorporate semantically meaningful business roles, which could have a diverse impact upon access decisions in such multi-domain collaborative business environments. We propose an Intelligent Role-based Access Control (I-RBAC) model that uses intelligent software agents for achieving intelligent access control in such highly dynamic multi-domain environments. The novelty of this model lies in using a core I-RBAC ontology that is developed using real-world semantic business roles as occupational roles provided by Standard Occupational Classification (SOC), USA. It contains around 1400 business roles, from nearly all domains, along with their detailed task descriptions as well as hierarchical relationships among them. The semantic role mining process is performed through intelligent agents that use word embedding and a bidirectional LSTM deep neural network for automated population of organizational ontology from its unstructured text policy and, subsequently, matching this ontology with core I-RBAC ontology to extract unified business roles. The experimentation was performed on a large number of collaboration case scenarios of five multi-domain organizations and promising results were obtained regarding the accuracy of automatically derived RDF triples (Subject, Predicate, Object) from organizational text policies as well as the accuracy of extracted semantically meaningful roles.

## 1. Introduction

Smart city applications need greater collaboration among companies, entrepreneurs and citizens [1]. These stakeholders can be from multiple domains and the citizens involved can perform multiple roles depending upon their collaborative application and organizational policy. It creates a dynamic multi-domain collaborative environment, which needs an effective intelligent access control [2] in order to protect information and resources from unauthorized entities. Many access control approaches have been proposed in last couple of decades but the significance of Role-Based Access Control (RBAC) [3] is inevitable in this regard. This model assigns permissions to resources based upon their roles and assigned tasks. However, applying this model in smart city applications’ multi-domain collaborative scenarios is highly challenging because it fails to adapt the dynamically changing information of the users and resources as well as being unable to automatically handle diversity of users’ multiple roles. In such an environment, discovering roles with business semantics as well as general classification of such business roles are unaddressed problems [4]. Moreover, smart city applications demand automatic identification of roles, permissions and objects from collaborating organizational textual policies. 

Our proposed I-RBAC model [5,6,7] is the first effort in finding solutions to these problems as well as combining the advantages of RBAC model with intelligent agents and ontology for finding semantically meaningful roles in highly dynamic multi-domain collaborative environments. These intelligent agents have learning capabilities and are adaptable to changing environments. This paper is focused on explaining our agent-based semantic role mining approach in multi-domain collaborative environments using the I-RBAC model. The main contributions involve derivation of organizational ontology from its policy text using already trained bi-directional LSTM. Later on, this automatically populated ontology is matched with our core I-RBAC ontology in order to extract unified semantic roles with business meaning. Our core I-RBAC ontology is already built based upon the tasks descriptions of business roles as per the Standard Occupational Classification (SOC) system [8]. Moreover, the bi-directional LSTM is also trained using this ontology and SOC system’s textual descriptions of tasks for business roles. The semantic role mining process is performed through intelligent software agents that utilize knowledge stored in ontologies.

The rest of the paper is organized as follows: Section 2 briefly introduces the intelligent role-based access control model through defining its main components and system architecture (interested readers may refer to our earlier published paper [5] for further detailed description of our proposed I-RBAC model and framework as well as its framework implementation).Section 3 summarizes the related work regarding existing extended models of RBAC, for multidomain collaboration, and their comparative analysis with our I-RBAC model to emphasize the importance of our I-RBAC model for multidomain collaborations in smart city applications. This section also summarizes existing work regarding semantic role mining in domain-specific RBAC, automated text to ontology derivation, ontology matching and alignment as well as the work where agents had been used in RBAC systems. Section 4 explains our proposed methodology for semantic role mining using the I-RBAC model. Section 5 explains its implementation through multiple agents. Section 6 discusses results and, finally, Section 7 concludes the paper and describes its limitations and future work.

## 2. Intelligent Role-Based Access Control (I-RBAC)

Our intelligent RBAC (I-RBAC) model is an extended version of the traditional RBAC model, and it has the capability of mining semantically meaningful business roles through intelligent software agents that can keep track of the dynamically changing environment, information sources available to the system and required access methods. These agents can activate new roles and can also change granted roles according to the new policy. The role hierarchy concept is bound to assigned tasks according to organizational hierarchy, which is different from the standard RBAC model. The main components of our proposed I-RBAC model are user (agent), business role, task role, set of tasks and permissions. These main components and their relationships are formally defined as given below.

The user (agent) can assimilate and interpret the environment changes independently. It acts according to the changing environment.

(1) $$Users(U_{Ag})=\{U_{Ag_{i}}|i=1,2,3,\ldots n\}$$

(2) $$\mathrm{Whereas}\;U_{ag}=\{A_{\mathrm{id}},\mathrm{ontology},\;\mathrm{communication},\;\mathrm{action},\;\mathrm{result}\}\;\forall U_{ag}\in U_{Ag}$$

The role is classified according to tasks assigned to each user. We categorize the Role as the Business Role (*BR_i_*) that is user’s exact job entitlement held in an organization. Task roles are a subset of the business roles set but are dynamic as per assigned tasks and named as Task Roles (TR). There is a many-to-many relationship between roles and agents.

(3) $$BusinessRoles(BR)=\{BR_{i}|i=1,2,3,\ldots n\}$$

(4) $$\mathrm{Whereas}\;\mathrm{br}=\{U_{ag_{1}},U_{ag_{2}},\ldots ,U_{ag_{n}}|U_{ag_{i}}\in U_{Ag}\}\;\forall br\in BR$$

The permission is an authorization to access system resources. It is the combination of actions performed on certain objects and is the power set of permissions associated with different tasks.

(5) $$\mathrm{Objects}(Obj)=\{Obj_{i}|i=1,2,3,\ldots n\}$$

(6) $$\mathrm{Operations}(Opr)=\{Opr_{i}|i=1,2,3,\ldots n\}$$

(7) $$Permissions(P)=\{P_{i}=Obj_{j}\times Opr_{k}|i=1,2,\ldots n\;\mathrm{and}\;j,k\in \{1,2,\ldots ,n\}\}$$

The task is a specific predefined set of tasks associated with a specific business role owned by different organizations.

(8) $$Tasks(T)=\sum_{n=1}^{N}T_{n}=T_{1}\cup T_{2}\cup \ldots \cup T_{N}=\{t|\exists n:(t\in T_{n})\}$$

The attribute can be related to users, roles and objects (resources).

(9) $$UserAttributes(Attr_{user})=\{Attr_{user_{i}}|i=1,2,\ldots n\}$$

(10) $$ObjectAttributes(Attr_{obj})=\{Attr_{obj_{i}}|i=1,2,\ldots n\}$$

(11) $$RoleAttributes(Attr_{role})=\{Attr_{role_{i}}|i=1,2,\ldots n\}$$

Task-Permission-Assignment (*TPA*) is defined as:

(12) $$TPA=\{\left(P,T,Attr_{Ojb},\;Attr_{Opr}\right)|PermissionPisassignedtoTaskT\}\subseteq P\times T\times Attr_{Obj}\times Attr_{Opr}$$

Role-Task-Assignment (*RTA*) is defined as:

(13) $$RTA=\{\left(BR_{i},\;T_{i}\right)|\;Task\;T_{i}\;is\;assigned\;to\;TaskRole\;TR_{i}\;\in \;BR_{i}\}\subseteq T\times BR$$

User-Role-Assignment (*URA*) is defined as:

(14) $$URA=\{\left(BR_{i},\;U_{Ag_{i}}\right)|BusinessRoleBR_{i}assignedtoUser(agent)U_{Ag_{i}}\;\subseteq BR\times U_{Ag}\}$$

The session is the time stamp allocated to a user while working under a certain role.

(15) $$Session\;\left(S\right)=\{S_{role}{}_{i}|role_{i}\in Brorrole_{i}\to T_{j}\;|\;i,j=1,\;2,\;3\ldots n\;\}$$

(16) $$Session-User_{Ag}:\;S\to U_{Ag}$$

The following, on the other hand, is a function that maps each session *S_i_* to a set of Task Roles

(17) $$TR\left(S_{i}\right)\subseteq \{BR|User\left(S_{i}\right),\;BR)\;\in URA\;\}$$

**Figure 1:**
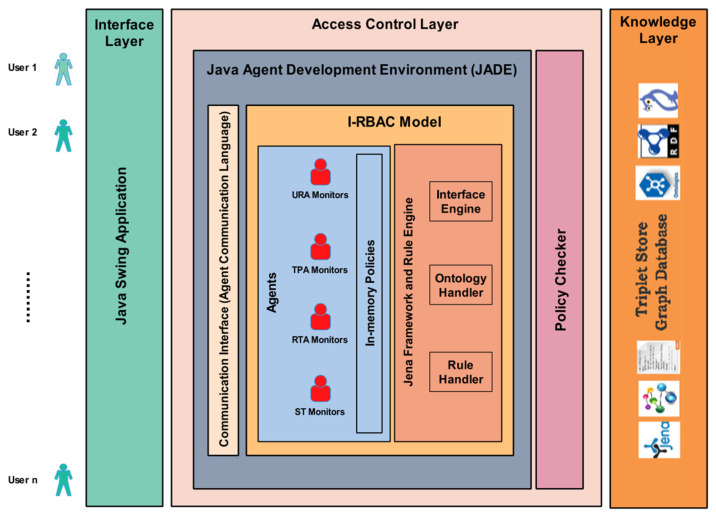
I-RBAC System Architecture.

I-RBAC system architecture is shown in Figure 1. The first layer provides an interface to the access control layer responsible for the overall security mechanism, based on role assignment to different users in multiple organizations. This layer consists of multiple intelligent agents and these agents are equipped with knowledge from the third layer, i.e., the knowledge layer. The knowledge, in this layer, is stored in the form of ontologies. The generic core I-RBAC ontology is based on real-world semantic business roles whose description is taken from Standard Occupational Classification (SOC) system, USA [8].

The organizational ontologies are automatically populated in the form of RDF schemas through text-based policies of those organizations. The intelligent agents use ontologies to classify roles, permissions, and objects. The interrelations between concepts and entities help to keep track of the roles and their assigned permissions on certain objects.

## 3. Related Work

In this section, some existing extended models of RBAC, for multidomain collaboration, are summarized and their comparative analysis with our I-RBAC model is given. Additionally, there are four possible dimensions of this research work and the related work in those dimensions is also summarized in separate subsections.

### 3.1. Existing RBAC Extended Models for Multidomain Collaborations

At present, different models and architectures of distributed computing over the internet have been developed for resource sharing and collaboration. The concept of virtual organizations has been introduced in these open, distributed computing environments to enable resource sharing and collaboration across different domains. However, these virtual organizations have to face the great challenge of security due to the dynamic and autonomous characteristics of participating domains [5]. To overcome this security challenge in multi-domain collaborative environments, access control models play an important role. The current literature review reveals that the RBAC model is the most adoptable model by different organizations regardless of their size, due to the simplicity of the model and ease of administration of relationships among users and permissions [9,10]. Although RBAC provides many benefits to organizations regarding the mapping of job functions to the RBAC roles and then encoding these mappings in the form of security policy, these security policies must align with the organizational structure and business needs [11].

There are limited works that tackle challenges for multidomain collaboration using an extended RBAC approach. The work in [12] proposed a policy integration framework for global coherent access control policy that is applicable for role-based access control in heterogeneous multidomain collaborative environments. The work reported in [13] employed role mapping for interaction among multidomain collaborative organizations. In [14], the authors proposed a hybrid access control mechanism using common ontology of the application domain. Similarly, the works in [15,16,17] used ontology-based semantic techniques for access control in multidomain collaboration scenarios. In [18], a fine-grained role-attribute access control is proposed combining the benefits of RBAC and attribute-based access control (ABAC). A domain-based RBAC model and architecture [19] is proposed for the adaptability of multidomain security requirements. The work in [20] presented a dynamic role-based access control in multidomain environments by utilizing context-based usage control access policies.

The literature reviewed for access control in multidomain collaboration showed that automated business role mining for access control in multidomain collaboration is an open research area. Table 1 summarizes several aspects of the above-mentioned existing work for access control in multidomain collaboration in order to highlight their limitations, and also gives a comparison with our proposed I-RBAC model.

### 3.2. Semantic Role Mining in Domain-Specific RBAC

Many role mining algorithms were proposed in domain-specific RBAC systems but few of them deal with business meaning. A role mining technique was proposed in [21] that derived roles based upon weights that were already associated with permissions as per their importance. Similarly, role mining algorithms were also proposed in [22] by optimizing policy quality metrics considering some primitive metrics, such as policy size and role interpretability, or compound metrics, which consist of both of these factors. Some role mining algorithms [23,24] were proposed that were based on machine-learning models, such as LDA and ATM. The generative RBAC models produced through these algorithms helped to resolve certain issues, for example, anomaly detection, identification of policy errors and policy reconciliation. These algorithms considered user attributes and their entitlements. Several semantic role mining approaches [9,10,11,25,26] were also proposed that created meaningful roles from a business point of view. These approaches used available business information in order to produce such roles. The authors of [27] conducted semantic role mining by handling dynamic access control policies in workflow systems particular to the healthcare domain. A genetic algorithm-based approach was proposed by [28] for solving the role mining problem in RBAC.

### 3.3. Automated Ontology Derivation from Text

Ontology derivation from text is proposed, by many researchers, using the LSTM deep neural network. The technique proposed by [29] used LSTM to create RDF schema from simple text using DBpedia ontology. In [30], LSTM was again used to create ontology for the physics domain by converting the text of a physics book. The research in [31] used bidirectional LSTM for proper word choice, based on its sentential context, in a domain-specific scientific writing task as well as a general-purpose writing task. In [32], stock market trend classification is conducted through text data by using LSTM, which automatically populates ontologies from text data of the stock market.

### 3.4. Ontology Matching and Alignment

In [33], ontology matching techniques are categorized as classical and advanced techniques. Both of these techniques have been already applied in multiple applications including e-learning [34], natural language processing [35], biomedical data [36], etc. Classical techniques, such as those described in [35,36,37], employ old matching mechanisms to perform ontology matching based on prior results. Such techniques are unable to deal with a large number of data properties (i.e., large-scale data) and are less efficient in accuracy but have the advantage of less time consumption. Advanced techniques such as those described in [38,39] employ advanced algorithms, for example, hybrid evolutionary algorithms. Such techniques are complex and more time consuming but have greater accuracy and work better on large scale data.

### 3.5. Agents Used in RBAC

To the best of our knowledge, no work has been found in the literature that used intelligent agents in any extended RBAC model for multidomain collaboration. However, a few research works found where agents were used for domain-specific RBAC applications. The research conducted in [40] proposed a method for role assignment to mobile agents for distributed environments. They proposed a simple public key infrastructure with RBAC for trust management. A multi-agent system was proposed by [41] to access distributed health care data using middle facilitator agent. The main contribution of [42] was to guarantee a secure communication channel between health institutions by means of a strong access control for mobile agents. The work of [43] proposed ontology for task representation to enhance agent coordination and collaboration through reasoning over tasks. An approach based on agent coordination context was proposed in [44] for RBAC-MAS infrastructure. Dynamic role adaptation by the mobile agent was proposed in [45], introducing adaptive mobile agents for fault tolerance in the running system.

## 4. Proposed Methodology of Semantic Role Mining

Our semantic role mining methodology consists of following three modules:

1. Automated population of organizational ontology from policy text;
2. Matching organizational ontology with core I-RBAC ontology;
3. Ontology-based semantic role mining through intelligent agents.

### 4.1. Policy Text to Ontology Derivation

**Figure 2:**
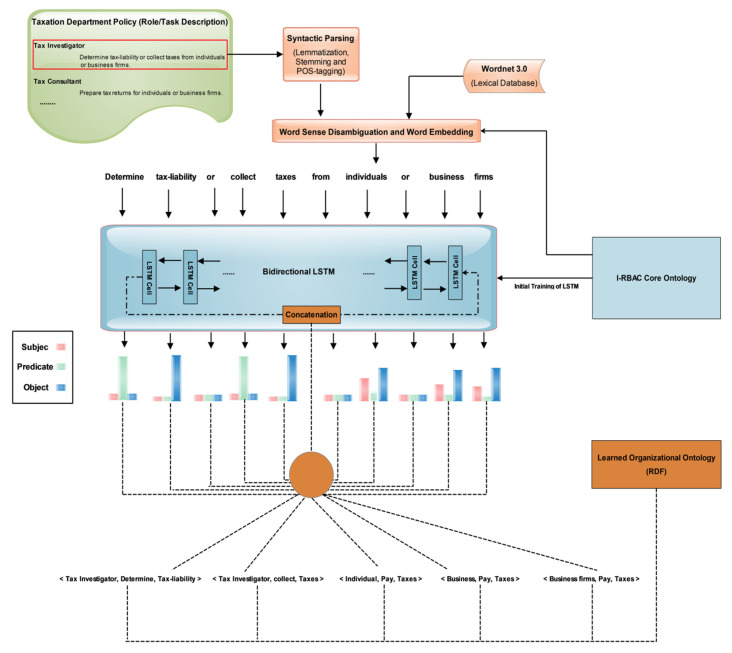
Automated population of organizational ontology from policy text.

The recent successes of neural language machine translations used by [29,30,31,32,46] convinced us to use Word Embedding (Word2Vec) and LSTM for the solution of our problem of converting textual policies into structural knowledge. The automated population of organizational ontology and its matching is shown in Figure 2.

First, organizational textual policy is preprocessed through POS (Part-of-Speech) tagging using the “CoreNLP API”, then word sense disambiguation is performed on this text using Wordnet 3.0, which is a lexical database of words, and it contains semantic relations between words. Word embedding is also performed on POS tagged text using the Word2Vec technique. Here, cosine similarity is measured among all words and their synonyms. After that, the output is fed to bi-directional LSTM (Bi-LSTM) [47] for extracting structured knowledge in the form of triples (subject, predicate, object). The reason for choosing Bi-LSTM is that it outperforms other models in such problems as it uses two LSTMs that increase the information to network about each sentential word’s context. It helps in finding the context of each word more effectively by knowing the words that immediately follow and precede it in the sentence. Figure 2 illustrates the whole process of automated population of organizational ontology from policy text.

Initially, we made our core I-RBAC ontology in Protégé. This ontology is made through real-world semantic business roles whose task description is taken from the Standard Occupational Classification (SOC), USA [8]. In addition to describing the tasks by each occupational role, this dataset also provides the hierarchical relationships among roles which make it ideal for building ontology from this textual description.

Later in the process, we trained a bidirectional LSTM deep neural network with the help of our core I-RBAC ontology’s concepts and word embedded vectors of corresponding textual description in a SOC dataset of around 1400 business roles from nearly all domains. The dataset is split into an 80/20 (train/test) data ratio.

**Figure 3:**
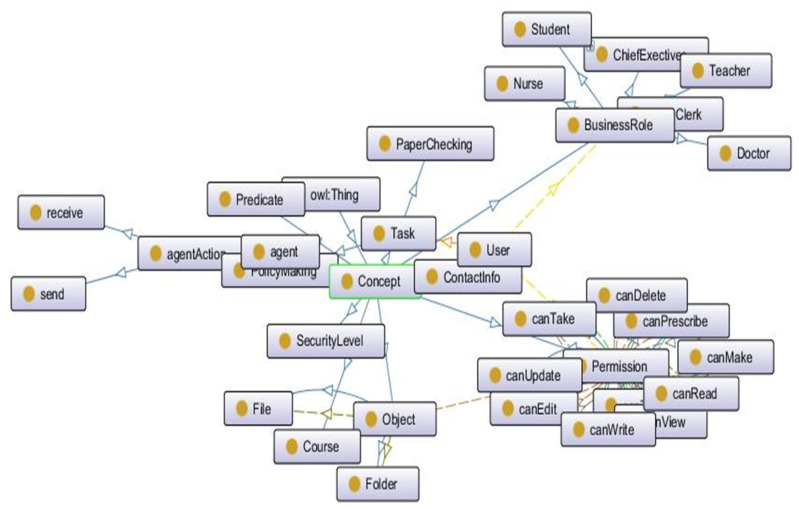
Snapshot of a part of core I-RBAC ontology.

In addition to roles and tasks, we also added general concepts of resources (objects), permissions, policies, agents and actions. This ontology also describes the interrelations among these entities and concepts. One of the snapshots of a part of our core I-RBAC ontology is shown in Figure 3.

**Figure 4:**
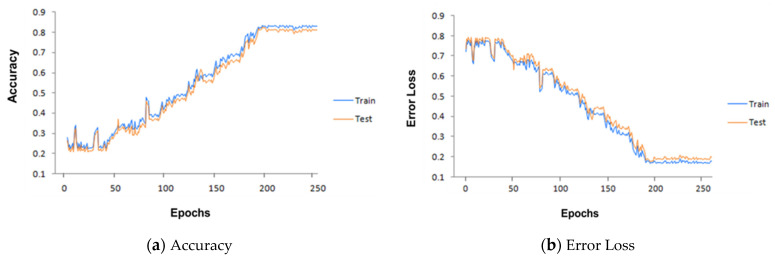
Our Bi-LSTM model performance as epochs increase.

Our bidirectional LSTM has 128 hidden layers. It is trained through our core I-RBAC ontology and textual data in the form of sentences. The textual data of the SOC list describes real world business roles, including their tasks and the hierarchical relationships among them. During training, the Bi-LSTM learned the sentential context of roles in the provided text and mapped textual descriptions with corresponding concepts provided through core I-RBAC ontology. First, the textual description is passed on to Word2Vec, which embeds words in vector form and, later on, those vectors are passed to Bi-LSTM as input, whereas the corresponding business role and its hierarchical relationships with other roles and objects are also passed to Bi-LSTM for training purposes. Such information is obtained from core I-RBAC ontology. Our Bi-LSTM worked best with the Adam Optimizer, with training parameters as given in Table 2. It attains an accuracy of around 83% in 200 epochs. The performance of the Bi-LSTM model is explained through Figure 4 with the help obtained graphs of accuracy and error loss along with increasing epochs.

**Figure 5:**
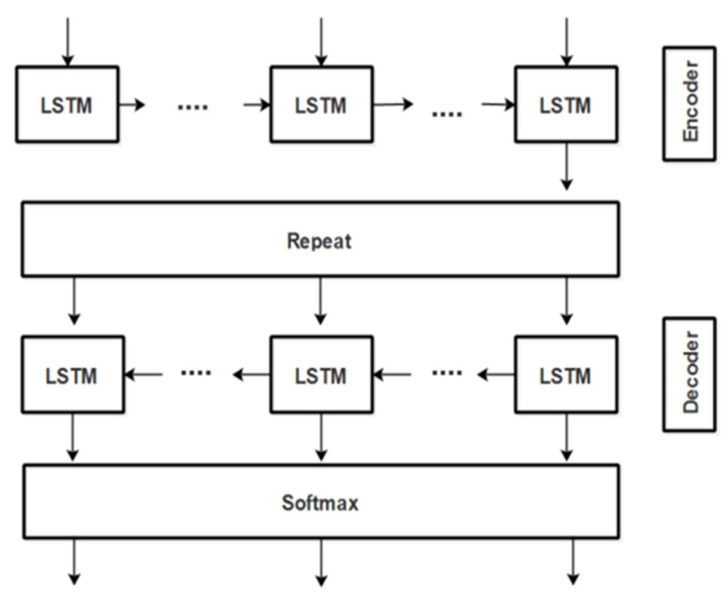
Encoder–decoder functionality of our Bi-LSTM.

The encoder–decoder functionality in our Bi-LSTM works best for identifying the triples because Bi-LSTM is capable of remembering past and future observations and, hence, remembering fine sentential context for concepts present in the text. The accuracy of extracted RDF triples subsequently enhances accuracy for identifying correct semantic roles from the dynamically available policy text. This bidirectional LSTM consists of two different LSTMs, forward and backward: one for encoding the input into embedded vector and the other for decoding the embedded vector to an output sequence. This model helps in preserving information both from previous and subsequent sequential contexts. In addition, such encoder–decoder architecture is useful in scenarios, like ours’, where the lengths of input and output sequences are not equal. Moreover, as our task is to label each word with subject/object/predicate tag so it is the best choice for our problem. Figure 5 shows this encoder–decoder functionality of our bidirectional LSTM.

Each LSTM network uses multiple sigmoid gates that control the information flow within the model. Our model also employed layer normalization (i.e., L_norm_) to prevent neurons from saturation via keeping their inputs centered. This is achieved by calculating the mean and standard deviation of the inputs and normalizing them accordingly at each time step. The complete LSTM equations that computed at each time instance t can be represented as follows.

(18) $$i\left(t\right)=\sigma [L_{\mathrm{norm}}(W_{\mathrm{ix}}\;X\left(t\right);\alpha_{i},\beta_{i}+L_{norm}\left(W_{ih}\;O\left(t-1\right);\alpha_{i},\beta_{i})\right]$$

(19) $$F\left(t\right)=\sigma [L_{\mathrm{norm}}(W_{\mathrm{Fx}}\;X\left(t\right);\alpha_{F},\beta_{F}+L_{norm}\left(W_{Fh}\;O\left(t-1\right);\alpha_{F},\beta_{F})\right]$$

(20) $$g\left(t\right)=\tan h[L_{\mathrm{norm}}(W_{\mathrm{gx}}\;X\left(t\right);\alpha_{g},\beta_{g}+L_{norm}\left(W_{gh}\;O\left(t-1\right);\alpha_{g},\beta_{g})\right]$$

(21) $$\rho \left(t\right)=\sigma [L_{\mathrm{norm}}(W_{\rho x}\;X\left(t\right);\alpha_{\rho },\beta_{\rho }+L_{norm}\left(W_{\rho h}\;O\left(t-1\right);\alpha_{\rho },\beta_{\rho })\right]$$

where input i(t) is the input gate, F(t) is forget gate, g(t) is modulation gate and σ is the function, whose output within interval [0, 1], helps in remembering or forgetting as its 0 value will cause complete forgetting and its 1 value will cause complete retention of the information. Hence, with the help of this σ function, the forget gate *F*(t) filters the current information in the cell state. We have not included bias in all four gates (i.e., Equations (18)–(21) because we have already used layer normalization. Finally, with the help of Equations (22) and (23), the output of each LSTM layer at time step t (i.e., O(t)) is calculated as:

(22) s(t)=g(t)⊛i(t)+s(t−1)⊛F(t)

(23) $$O\left(t\right)=\tanh[L_{\mathrm{norm}}\left(s\left(t\right);\alpha_{s},\beta_{s}\right]⊛\rho \left(t\right)$$

where ⊛ denotes element-wise multiplication, W_*x_ represents input weight matrices, W_*h_ represents recurrent weight matrices and $\alpha_{*}$, $\beta_{*}$, are trainable vectors used to fit the output distribution in order to normalize layers. Moreover, *tanh* is the hyperbolic tangent function, σ is the sigmoid function, and $L_{norm}$ is the layer normalization function. These three functions are represented by Equations (24)–(26), respectively.

(24) $$\tanh(x)=\frac{{}^{e^{x}-e^{-x}}}{{}^{e^{x}+e^{-x}}}$$

(25) $$\sigma (x)=\frac{1}{{}^{1+e^{-x}}}$$

(26) $$L_{norm}(z;\alpha ,\beta )=\frac{z-\mu }{{}^{\sigma }}⊗\alpha +\beta$$

where, the sybmol µ in Equation (26) represents the mean value. The output gate O passes information to the next hidden states and hence, at each time step t, each hidden layer’s input i.e., *i*(t) gets the previous layer’s output, i.e., O(t − 1). The encoder and decoder LSTM networks shown above in Figure 5 are used to extract the features; the encoder identifies the general features and the decoder identifies the more specific features. For example, the encoder finds the word CEO and File as nouns in the encoder layer and distinguishes them as Subject and Object in the decoder layer. The output of the encoder-LSTM is fed to the decoder-LSTM and then this output is passed through the distributed softmax layer that calculates the raw output as probabilities. Then, the model is trained through the cross-entropy loss function that measures the divergence of probability estimates (output of softmax layer with respect to the true labels). The final output of our trained bidirectional LSTM is RDF schema in the form of set of triples (subject, predicate and object) from dynamically loaded organizational policy text. The algorithm of triple extraction from LSTM is given as Algorithm 1.

**Algorithm 1**: Triple Extraction. 1: **Input:** policy text corpus (C_PT_) 2: **Output**: RDF 3: **begin** 4: load (text, onto) 5: cleantxt = preprocess(txt) 6: cleanOnto = preprocess(onto) 7: textDictionary = Word2Vec(cleantxt) 8: ontoDictionary = Word2Vec(cleanOnto) 9: encoder LSTM (sequenceClassifier) 10: decoder LSTM (sequenceClassifer) 11: new_rules{ } = infer(data); 12: **end**

### 4.2. Ontology Matching

After extraction of the organizational schema (i.e., RDF) by LSTM, this schema automatically populates the organizational ontology, which will be further matched with core I-RBAC ontology in order to extract unified business roles through agents. Therefore, ontology matching and mapping are very important tasks during the semantic role mining process. Sometimes, the ontology matching term is alternatively used as semantic matching, which refers to computing relationships between the nodes of two different graphs.

To find the equivalence between two ontologies, there is a need to understand the semantics of relations between concepts of those two ontologies. The equivalence relations are synonym relationships showing semantic similarity between two concepts. It is a binary relation between two terms. Two terms may be syntactically equal; however, their semantic equality depends upon the context in which they are used. For example, there are different roles named “manager” either in a bank or in any other company. The term “manager” refers to a role in an access control policy scenario, but, to identify its equivalence with some role in I-RBAC, it is necessary to find its semantic equivalence based upon its tasks.

**Figure 6:**
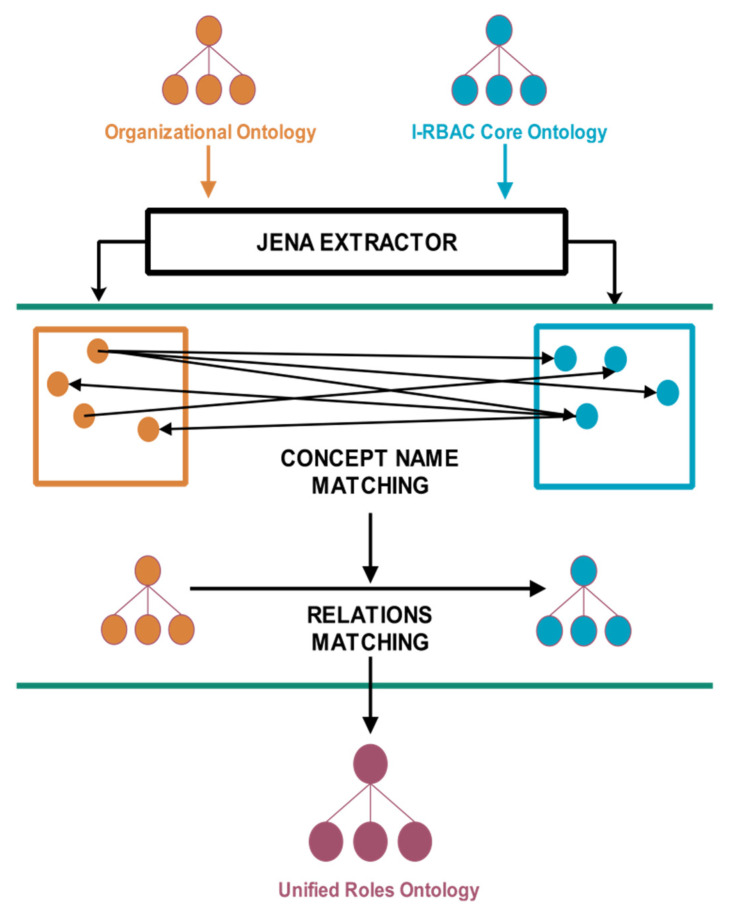
Ontology matching paradigm.

In distributed heterogeneous systems, there is a need for automation in order to ensure effective interoperability. Therefore, we proposed a multi-agent based ontology mapping technique to achieve dynamicity, adaptability and scalability in our I-RBAC framework. We have used word embedding equivalence, which finds semantic similarity between two concepts and, in our approach, one-to-one cosine similarity (given in Equation (27)) is measured for all pairs of concepts, whereas, in that pair, the one concept is taken from the first ontology and the other concept is taken from the second ontology. The ontology matching paradigm is visually illustrated in Figure 6.

(27) $$\cos\left(a,b\right)=\frac{a.b}{\left|a\right|\;\left|b\right|}$$

Another task is to find semantic similarity among object properties (relations). It is relatively difficult because there may be combinations of different words with some prefixes, such as is-a, has-a, etc. Thus, the core concept is considered in this case and tries to find the first verb by applying POS tagging using CoreNLP API and, at the end, synonyms are found from WordNet and Jaccard similarity (given in Equation(28)), which is calculated to find the semantic similarity between two object properties.

(28) $$\mathrm{jaccardSim}\left(s,t\right)=\frac{s\;\cap \;t}{s\;\cap \;t}$$

### 4.3. Semantic Role Mining

The core I-RBAC ontology is the backbone of the whole I-RBAC model as well as being of utmost importance in the semantic role mining process through agents. A snapshot of the partial core I-RBAC ontology is shown in Figure 3. The basic components of this ontology are roles, task, objects and permissions. Roles are the owners of the resources, objects are resources, and permissions are access rules to perform certain actions on a specified resource to accomplish a given task. The following tuple represents the general form of the access rule.

(29) $$AccessRule\equiv <R_{i},T_{j},O_{k},P_{r}>$$

where *R_i_* is the target role, *T_j_* represents the task to be performed, *O_k_* represents a certain resource (object) to be accessed and *P_r_* is the type of permission (e.g., read, write etc.) along with the states of the permissions. The interrelations among all these concepts determine the role of the user. In our I-RBAC architecture, semantic business roles are mined through JADE agents. These agents are capable of understanding ontology and ontological information is stored and communicated among agents in the form of java objects. For communication among agents, our I-RBAC architecture used FIPA-ACL, which provides a common language (sharing common vocabulary) for communication among agents. Our utilized Jena framework provides support for RDF and OWL, and it proved best in mining roles through the Jena Inference Engine (JIE) or Pellet reasoner. JIE also supports ontology population through additional RDF assertions based upon certain SWRL rules. Finally, the Jena reasoner leads the semantic role mining agent to infer business roles from the knowledge graph with the maximum number of permissions based upon SWRL rules and SPARQL queries. This whole mechanism is described below in Algorithm 2.

**Algorithm 2**: Semantic Role Mining. 1: **Input**: rdf_graph, pre_rules{ }, environment E 2: **Output**: R={t{ }p{ }, *rdf_graph* 3: **begin** 4: model M=loadOnto (*rdf_graph*) 5: **while** (!EOM) **do** 6:  concepts{ } = M.retrieveClass( )     resources{ } = M.retrieveDataProperty( )     relations{ } = M.retreivePredicate( ) 7: **end while** 8: data{{},{},{}} = combine (concept, resources, relations) 9: new_rules{ } = infer (data) 10: agent_onto = learn (pre_rules{ }+ new_rules{ }) 11: updated_rule = infer (agent-onto) 12: rdf-graph = construct (updated_rule, concepts{ }, resources{ }, relations{ }) 13: role = getRole (agent-onto) 14: user = setRole (t{ }, p{ }) 15: return *rdf-graph* 16: **end**

## 5. Agent-Based Implementation

As already described above, the I-RBAC framework uses a multi-agent paradigm for implementation of the whole role mining process. Such implementation helps to mine meaningful business roles in an automated way and is also applicable in highly dynamic collaborative environments consisting of organizations from heterogeneous/multiple domains. Hence, our implementation achieves the goals of dynamicity, adaptability and scalability in such environments.

**Figure 7:**
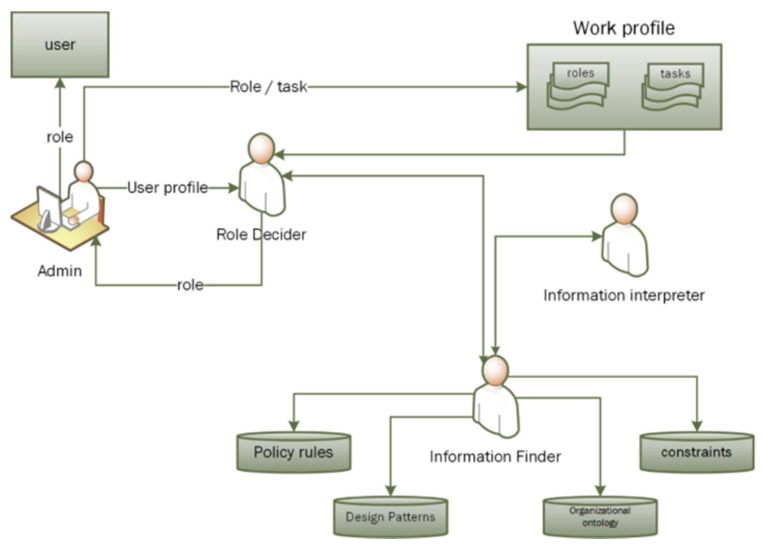
Multi-agent paradigm for role assignment.

Software agents are autonomous in classifying, analyzing and searching knowledge from various sources. As stated above, the knowledge is stored in the form of ontologies in the I-RBAC framework. Agents utilize this knowledge to fulfill their responsibilities. The multi-agent paradigm for role assignment is shown in Figure 7.

It consists of the “*Admin*”, who is responsible for fetching the organizational text policy from the organizational repository and, finally, assigning roles to users (at the end of the role mining process); the “*RoleDecider*” agent is responsible for deciding roles based upon the information it retrieved; the “*InformationInterpreter*” agent is responsible for inferring rules in order to mine roles; and the “*InformationFinder*” agent is responsible for searching knowledge from several ontologies. This multi agent paradigm is implemented through JADE agents and agents communicate through FIPA-ACL, which functions as a common language among them. Our utilized JENA framework provides enough support to search, retrieve or augment knowledge in ontologies that are utilized by the “InformationFinder” agent. Lastly, the “*InformationInterpreter*” and “*RoleDecider*” agents use the Jena Inference Engine (JIE)/Pellet reasoner for reasoning, inferring and deciding the best possible business role for the user.

## 6. Results and Discussion

The multi agent simulations of I-RBAC architecture are implemented on a standard desktop PC with an Intel core i3-6100 CPU, NVIDIA GeForce GTX-1070 GPU, 16 GB RAM and 1TB hard disk. This system has a 64-bit Windows10 operating system. All simulations are performed in the Eclipse IDE for the Java Development Environment (JADE, JENA with Alignment API, Neo4J database). The Protégé environment is used for ontology modeling. The SOC [8] dataset of task description is used for the construction of I-RBAC ontology. Evaluation results are obtained by providing different organizations’ policy texts. The SPARQL query language is used to run different queries. The performance is based on the OntoClean [48] recommendations, i.e., consistency and completeness of ontologies. The consistency and completeness of the ontologies are evaluated using Pellet and the JENA Inference Engine (JIE). Pellet and JIE, alone as well in combination, were used for reasoning on ontology knowledge using pre-defined SWRL rules.

**Figure 8:**
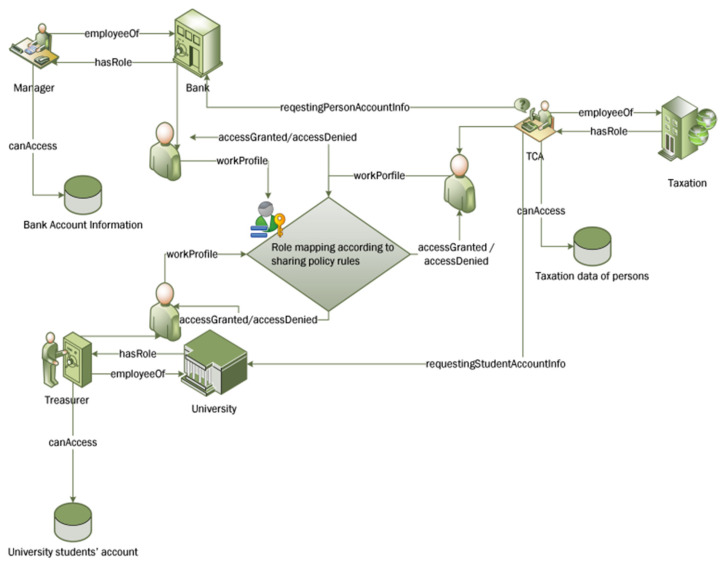
Collaborative environment used in experimental scenarios.

The experiments were run through simulating collaborative case scenarios of information sharing among five organizations belonging to three different domains (two Universities, two Banks and a Taxation department). Overall, 113 roles were created from these organizations. The same user could be assigned different roles at different stages depending on collaborative tasks at that particular time, and his/her organizational policy text defining tasks and permissions for each role. One of the collaboration scenarios is illustrated in Figure 8. For example, in this illustrated scenario, a Tax Calculating Officer (TCO) has an account in the Bank and his children are in the University, and he also has a tax account in the taxation office where he works. So, this person wanted access to different resources at different time intervals and he was assigned different roles as per predefined rules and structured knowledge stored in ontologies.

**Figure 9:**
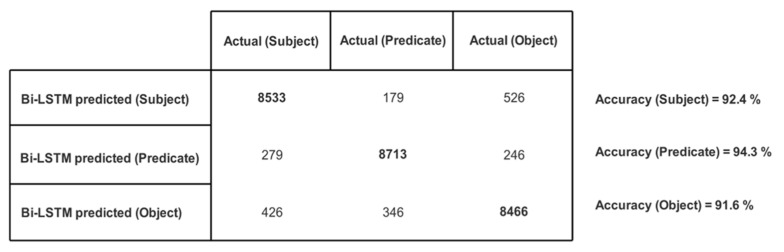
Confusion matrix showing no. of actual and Bi-LSTM predicted subjects/predicates/objects.

**Figure 10:**
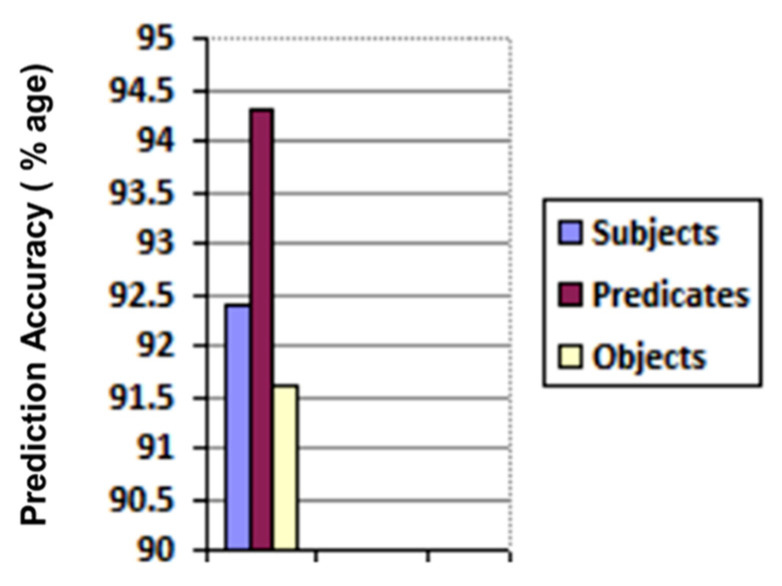
Prediction accuracy of subject/predicate/object in bi-LSTM returned triples.

We created 175 different collaboration case scenarios among 113 possible semantic business roles in the above-mentioned five organizations. The participating organizations shared their text policies defining tasks and permissions for business roles. These text policies were given to our trained Bi-LSTM, which returned structured knowledge as triples (subject, predicate, object). There was a total of 9238 returned triples. The correctness of each entity (i.e., subject, predicate, and object) in every triple is checked, and the number of actual and Bi-LSTM predicted entities are recorded in the confusion matrix given in Figure 9 and their accuracies are illustrated in Figure 10.

Furthermore, there were 7963 triples (out of 9238 returned triples) where all three entities (i.e., subject, predicate and object) were accurately predicted. This gives an overall prediction accuracy of 86.2% for Bi-LSTM returned triples.

**Figure 11:**
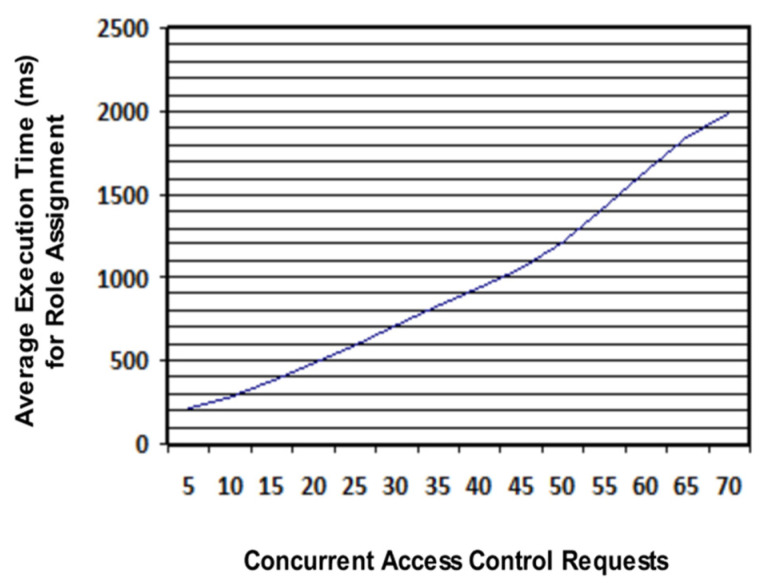
System response time in assigning roles for concurrent access control requests.

In each collaborative scenario, different access control requests were made by users. In total, 3260 access control requests were made for role assignment. Collaboration scenarios are designed in a way that concurrent access control requests are gradually increased from 5 to 70 and the average response time of system, for assigning roles, is recorded. It was observed that our system responded within 2 s for a maximum of 70 concurrent access control requests, as illustrated in Figure 11. Hence, it proves the high scalability of our system.

**Figure 12:**
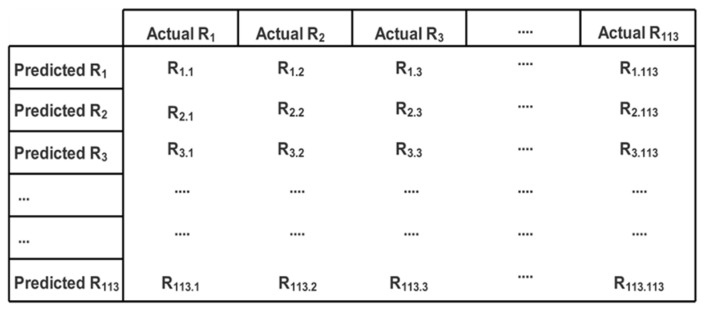
Confusion matrix representing number of predicted roles.

For every access control request, the verification of results is performed in order to measure the correctness of the semantic role mining process. The correctness is measured in terms of the true identification of roles. This measurement is represented through a confusion matrix given in Figure 12. In this confusion matrix, each R_i.j_ represents the number of predictions of role as R_i_, whereas it was actually R_j_.

The three metrics used are precision, Recall and F1 measure. Precision tells us how sure we are about the roles of our identified “true positives”, and recall gives us an idea of how sure we are about not missing any positives. The F1 measure gives the harmonic mean of precision and recall. All these three metrices are calculated for each role as follows:

(30) Precision of Ri (i.e., PRi)=Ri.i∑j=1TR Ri.j

where TR represents Total no. of Roles (which is 113 in our case)

(31) Recall of Ri (i.e., RRi)=Ri.i∑j=1TR Rj.i 

(32) F1 of Ri(i.e., F1Ri)=2∗ PRi∗ RRi(PRi+RRi)

Their average scores are calculated as follows:

(33) Average Precision (i.e.,AP)=∑i=1TR PRiTR 

(34) Average Recall (i.e.,AR)=∑i=1TR RRiTR 

(35) Average F1 (i.e.,AF1)=∑i=1TR F1RiTR 

Since the 3260 access control requests do not include a balanced number of actual roles and predicted (mined) roles so the best metric to calculate is the “weighted average” for all three metrices, which are calculated as follows:

(36) Weighted Average Precision (i.e.,WAP)=∑i=1TR((∑j=1TRRj.i)∗PRi)TACR 

where TACR represents Total no. of Access Control Requests (which is 3260 here)

(37) Weighted Average Recall (i.e.,WAR)=∑i=1TR((∑j=1TRRj.i)∗RRi)TACR

(38) Weighted Average F1 (i.e.,WAF1)=∑i=1TR((∑j=1TRRj.i)∗F1Ri)TACR 

**Figure 13:**
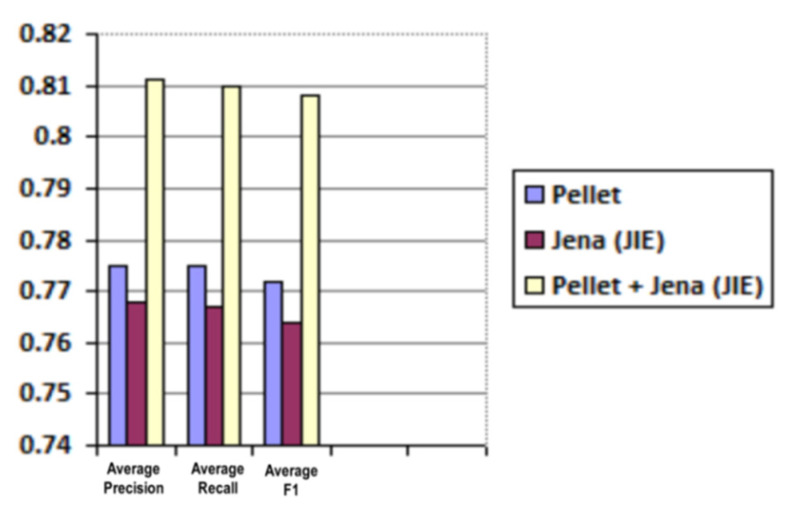
Predicted roles evaluation (average precision, recall and F1).

**Figure 14:**
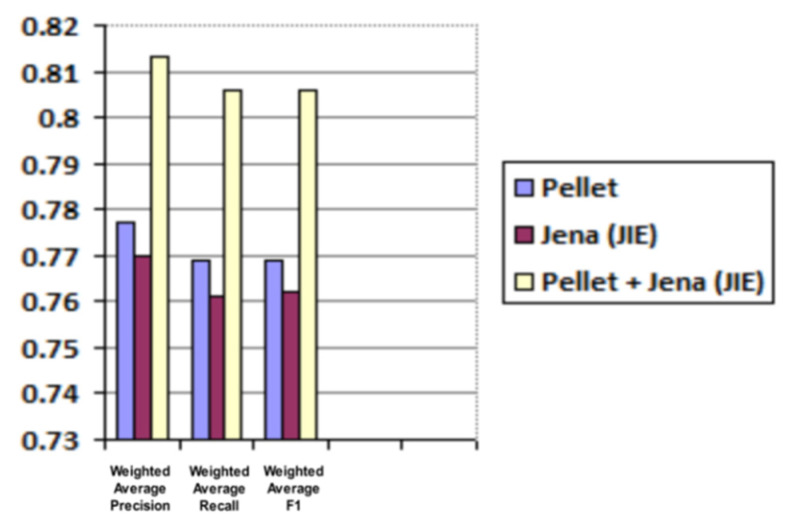
Predicted roles evaluation (weighted average for precision, recall and F1).

Average and weighted average results for precision, recall and F1 are shared in Table 3 and Table 4 and are graphically illustrated in Figure 13 and Figure 14, respectively.

The obtained results show good accuracy of mined roles as well as reasonable system response time in assigning roles while handling a sufficient number of concurrent access control requests. Hence, overall, it proved the effectiveness and scalability of our methodology as the first attempt at automated business role mining for access control in dynamic multidomain collaborative applications.

## 7. Conclusions and Future Work

In this paper, a novel agent-based semantic role mining approach is proposed that is workable in highly dynamic multi-domain collaborative scenarios of smart cities and discovers roles with business semantics. Its implementation involves automatic derivation of organizational ontology from its policy text with the help of a bi-directional LSTM that is already trained through our core I-RBAC ontology of real-world semantic business roles, whereas the core ontology was built based upon the tasks descriptions of business roles as per Standard Occupational Classification (SOC) system [8]. The proposed approach achieves the ideal goals of dynamicity, adaptability and scalability as it is adaptable to new organizational policies as well as being able to mine business roles in highly dynamic multi-domain collaborative environments. The promising experimentation results were obtained regarding accuracy of automated derived RDF triples (Subject, Predicate, Object) from text policies as well as predicted semantic business roles for users.

In the future, we intend to implement and test this model on further large-scale practical scenarios and for more access control requests. Moreover, the Standard Occupational Classification (SOC) system covers around 1400 business roles at the moment and these roles may increase in future; resulting more fine-tuned core I-RBAC ontology which will ultimately increase the accuracy of automatically mined semantic business roles.

## Figures and Tables

**Table 1:** Comparison of our I-RBAC model with existing extended RBAC models for multi-domain collaboration.

Ref.	Role Hierarchy	Attributes	Semantic Techniques and Technology	Machine Learning Techniques	Business Roles	Intelligent Agents
[12]	☑					
[13]	☑	☑				
[14]	☑		☑			
[15]		☑	☑			
[16]			☑			
[17]			☑			
[18]	☑	☑				
[19]		☑				
[20]	☑	☑				
Our Model	☑	☑	☑	☑	☑	☑

**Table 2:** Hyperparameter values for training of our Bi-LSTM.

Parameter	Value
No. of layers	128
No. of neurons in LSTM layer	100
Dropout rate	0.2
Batch size	64
No. of epochs (*for minimum loss error*)	200
Initial learning rate	0.005
Optimization Method	ADAM

**Table 3:** Predicted roles evaluation (average values for precision, recall and F1).

Reasoner	Average Precision	Average Recall	Average F1
Pellet	0.775	0.775	0.772
Jena (JIE)	0.768	0.767	0.764
Pellet + Jena (JIE)	0.811	0.81	0.808

**Table 4:** Predicted roles evaluation (weighted average values for precision, recall and F1).

Reasoner	Weighted Average Precision	Weighted Average Recall	Weighted Average F1
Pellet	0.777	0.769	0.769
Jena (JIE)	0.77	0.761	0.762
Pellet + Jena (JIE)	0.813	0.806	0.806

## Data Availability

The SOC dataset used in this study is available online [8].
